# Efficacy and safety of intravesical dimethyl sulfoxide treatment for patients with refractory Hunner‐type interstitial cystitis: Real‐world data postofficial approval in Japan

**DOI:** 10.1111/iju.15320

**Published:** 2023-10-11

**Authors:** Yoshiyuki Akiyama, Aya Niimi, Akira Nomiya, Satoru Taguchi, Yuta Yamada, Yusuke Sato, Taketo Kawai, Daisuke Yamada, Haruki Kume, Yukio Homma

**Affiliations:** ^1^ Department of Urology, Graduate School of Medicine The University of Tokyo Tokyo Japan; ^2^ Department of Urology New Tokyo Hospital Matsudo Chiba Japan; ^3^ Department of Urology Kanto Rosai Hospital Kawasaki Kanagawa Japan; ^4^ Department of Urology Teikyo University School of Medicine Tokyo Japan; ^5^ Department of Interstitial Cystitis Medicine Kyorin University School of Medicine Tokyo Japan

**Keywords:** bladder pain syndrome, dimethyl sulfoxide, DMSO, Hunner, IC/BPS, interstitial cystitis

## Abstract

**Objectives:**

To examine real‐world data regarding intravesical dimethyl sulfoxide (DMSO) therapy after official approval as a treatment for Hunner‐type interstitial cystitis (HIC) in Japan.

**Methods:**

This single institution, retrospective observational study was conducted between 2021 and 2022 to evaluate the outcomes of 30 patients with refractory HIC who received intravesical DMSO therapy according to the approved standardized regimen: administration of DMSO every 2 weeks for a total of 12 weeks. Treatment outcomes were evaluated using a 7‐graded global response assessment scale, O'Leary and Sant's symptom and problem indices (OSSI/OSPI), the overactive bladder symptom score (OABSS), an 11‐point pain intensity numerical rating scale, quality of life (QOL) score, and frequency volume chart variables. Related complications were also documented.

**Results:**

The response rates at 2, 4, 6, 8, 10, and 12 weeks were 36.7%, 43.3%, 53.3%, 60.0%, 70.0%, and 70.0%, respectively. Compared with baseline, OSSI/OSPI, pain intensity, urinary frequency, and the QOL score improved significantly from 4 weeks of treatment. The OABSS score and functional bladder capacity also showed a tendency toward moderate improvement, but the difference was not significant. The mean duration of symptom relapse after termination of treatment was 6.4 ± 3.9 months. No patients discontinued treatment due to adverse events, although acute bladder irritation during infusion was noted in 21 patients (70%), which disappeared within 3 days.

**Conclusions:**

This study verifies the safety, moderately durable efficacy, and tolerability of the standard intravesical treatment with DMSO for HIC in Japan.

**Abbreviations**
AE: adverse event
DMSO: dimethyl sulfoxide
ESSIC: International Society for the Study of IC/BPS
GRA: global response assessment
HIC: Hunner‐type interstitial cystitis
IC/BPS: Interstitial cystitis/bladder pain syndrome
NSAIDs: nonsteroidal anti‐inflammatory drugs
OABSS: overactive bladder symptom score
OSSI/OSPI: O'Leary and Sant symptom index/O'Leary and Sant problem index
PDE5: phosphodiesterase type 5
PSL: prednisolone
QOL: quality of life
SD: standard deviation

## INTRODUCTION

Interstitial cystitis/bladder pain syndrome (IC/BPS) is an intractable, devastating urological disorder of unknown etiology, characterized by persistent bladder pain accompanied by lower urinary tract symptoms such as urinary frequency and urgency.^1^ IC/BPS is categorized into two subtypes, Hunner‐type interstitial cystitis (HIC) and BPS, based on the presence or absence, respectively, of Hunner lesions on cystoscopy.^1^ Past studies have shown that HIC is a distinct, immune‐mediated chronic inflammatory disease of the urinary bladder with a possible autoimmune basis, whereas BPS is a non‐inflammatory disorder with little evidence of bladder pathology; the latter may be associated with bladder‐beyond etiologies such as systemic neurophysiological/endocrine dysregulation.^1^, ^2^, ^3^, ^4^ Thus, treatment strategies for IC/BPS should be undertaken in a subtype‐directed manner; for example, anti‐inflammatory/immunomodulatory approaches for HIC and systemic neuromodulation approaches for BPS.^4^ Although there are no curative treatments for IC/BPS, dimethyl sulfoxide (DMSO) has been used widely and is known to be effective at relieving symptoms.^5^, ^6^, ^7^, ^8^, ^9^, ^10^, ^11^, ^12^, ^13^ Studies show that one of the major action mechanisms of DMSO is the suppression of overactive immune responses by reversing upregulation of pro‐inflammatory and nociceptive genes such as interferon‐γ, interleukin‐6, tumor necrosis factor‐α, nerve growth factor, and MCP‐1, in conjunction with impairing activated effector T cells.^14^, ^15^ Given these anti‐inflammatory effects of DMSO, it was not unexpected that a recent multicenter, placebo‐controlled, randomized study of intravesical DMSO treatment for IC/BPS in Japan reported a favorable treatment response in patients with HIC compared with those with BPS.^16^ Based on these results, the Japanese Ministry of Health, Labour, and Welfare officially approved intravesical DMSO therapy as a treatment for HIC in 2021. At the time of approval, the standard treatment regimen was also determined as follows: instillation of DMSO every 2 weeks for 12 weeks (a total of six treatments). Here, we examined postofficial approval, real‐world data from patients receiving the standard intravesical DMSO treatment for HIC at our tertiary referral center in Japan. We report the safety, moderately durable therapeutic efficacy, and tolerability of this standard treatment, which was particularly highlighted by significant pain relief and improved patients' quality of life (QOL).

## METHODS

### Ethics statement

This study, including the use of an opt‐out methodology to obtain informed consent, was approved by the Institutional Review Board of the University of Tokyo (approval no. 3124). Patients were informed about the study using generally accessible contact information, and written informed consent was obtained from all those who chose to take part. All procedures followed appropriate guidelines.

### Patients

The present study is a retrospective observational study conducted in April 2023, of a prospectively maintained database of patients with refractory HIC treated with a series of six intravesical DMSO instillations between July 2021 and November 2022. Diagnosis of HIC was based on the East Asian clinical guidelines,^1^ and on the International Society for the Study of IC/BPS (ESSIC) criteria.^17^ All patients had been treated with electrocautery of Hunner lesions, with concomitant bladder hydrodistension, at least once, with or without subsequent salvage oral prednisolone (PSL) or other intravesical treatments using heparin and alkalized lidocaine, or botulinum toxin type A (BoNT‐A), in conjunction with other conservative therapies (Table 1). DMSO therapy was offered when a patient's pain relapsed after endoscopic surgery with or without subsequent salvage therapies, and the pain could not be managed by a combination of analgesic agents. Diagnosis, endoscopic surgery, and all conservative treatments, including DMSO treatment, for all patients were performed by a single urologist (YA).

**TABLE 1:** Demographic and baseline characteristics of the study subjects.

No. (male/female)	30 (12/18)
Mean age (years)	68.3 ± 12.2 [40–88]^a^
Duration of illness (years)	7.8 ± 4.7 [3–25]
OSSI	15.6 ± 3.6 [4–20]
OSPI	12.7 ± 3.5 [1–16]
Pain intensity^b^	7.1 ± 1.6 [4–10]
OABSS	9.2 ± 3.2 [1–15]
QOL score^c^	5.4 ± 0.9 [3–6]
Daytime frequency	13.6 ± 5.7 [5–33]
Nocturia	4.7 ± 3.3 [1–17]
Average voided volume (mL)	92.5 ± 61.8 [20–280]
Maximum voided volume (mL)	138.3 ± 84.7 [30–400]
Previous treatments^d^	
Transurethral resection of Hunner lesions with bladder hydrodistension (no. of patients)	30
Time on average (range)	2.0 ± 1.2 [1–6]
Maximum bladder capacity at the last hydrodistension (mL)	395.4 ± 150.8 [150–650]
Intravesical treatment (no. of patients)	3
Heparin and alkalized lidocaine	2
Botulinum toxin type A	1
Oral prednisolone (no. of patients)	6
Medicines (no. of patients)^d^	
Opioids	16
Acetaminophen	15
NSAIDs	13
Beta‐3 adrenoceptor agonists	12
Anticholinergic agents	8
Alpha‐1 adrenoceptor antagonists	8
Suplatast tosilate	6
PDE5 inhibitor	6
Tricyclic antidepressant	5
Pregabalin	5
Cernitin pollen extract	4
Benzodiazepine	1

Abbreviations: DMSO, dimethyl sulfoxide; NSAIDs, nonsteroidal anti‐inflammatory drugs; OABSS, overactive bladder symptom score; OSSI/OSPI, O'Leary and Sant symptom index/O'Leary and Sant problem index; PDE5, phosphodiesterase type 5; QOL, quality of life.

^a^ Mean ± SD (range).

^b^ Assessed using an 11‐point pain intensity numerical rating scale: from 0 (“no pain”) to 10 (“the worst pain ever”).

^c^ Assessed on a 7‐grade QOL scale derived from the International Prostate Symptom Score, with 0 indicating “excellent” and 6 indicating “terrible.”

^d^ Counted duplicate when multiple treatments/medicines were used.

### Protocol for intravesical DMSO therapy

A total of 50 mL of 50% DMSO (containing 27 g of DMSO) was administered intravesically using a 12‐Fr urethral catheter and remained in the bladder for 15 min; this was repeated every 2 weeks for a total of 12 weeks (six instillations in total) and represented a single course of the standardized DMSO therapy. For patients with reduced functional bladder capacity (<50 mL per void) and severe infusion reactions (e.g., bladder/urethral irritation accompanied by reactive incontinence), the infusion volume was reduced to half (25 mL) and the dwell time was shortened to 5–10 min according to the severity of the symptoms. A diclofenac suppository was given 30 min before the DMSO injection to patients experiencing acute infusion reactions. When no therapeutic benefits or treatment failure were observed during treatment, and unless a patient demanded treatment cessation, a single course of DMSO therapy was completed (total of six treatments); this was based on evidence of a frequency‐dependent cumulative effect of intravesical DMSO treatment for HIC (shown in the above‐mentioned randomized study).^16^ Patients attended a follow‐up visit 1 month after termination of treatment (i.e., at 12 weeks of treatment), followed by visits every 3 months thereafter until symptom relapse. No other treatments (e.g., endoscopic surgery, oral PSL, or other intravesical treatments) were given in parallel with DMSO treatment, although additional use of analgesic agents such as opioids, acetaminophen, pregabalin, or nonsteroidal anti‐inflammatory drugs (NSAIDs) was allowed.

### Outcome assessment

Treatment response was evaluated using a global response assessment (GRA) questionnaire, which is a 7‐point symmetric scale scored as follows: markedly improved (+3), moderately improved (+2), slightly improved (+1), no change (0), slightly worse (−1), moderately worse (−2), and markedly worse (−3). Patients who rated treatment efficacy as better than +2 in the GRA were considered as responders. Likewise, symptom relapse after treatment for patients with slightly or better improved conditions after DMSO therapy was defined when patients rated their conditions as worse than −1 in the GRA. At every visit, patient symptoms were evaluated using the IC/BPS symptom scores, measured using the O'Leary and Sant's symptom index and problem index (OSSI/PI); an 11‐point numerical rating of pain intensity, with 0 indicating no pain and 10 indicating the worst pain ever; a 7‐grade QOL scale derived from the International Prostate Symptom Score, with 0 indicating excellent and 6 indicating terrible; and the overactive bladder symptom score (OABSS). Daytime and nocturnal urinary frequency and maximum and average voided volume were also documented. Demographic information, including age at commencement of DMSO therapy, duration of illness, previous treatments, and maximum bladder capacity measured at the last session of bladder hydrodistension at a pressure of 80 cm H_2_O under general anesthesia, was also documented. The author (YA) had access to information that could identify individual participants during or after data collection.

### Safety assessment

Adverse events (AE) and side effects occurring during DMSO treatment were monitored carefully. At every visit, all patients underwent urine analyses and were checked for physical manifestations, vital signs, and urinary tract infections.

### Statistical analysis

Multiple comparisons between the symptom parameters at each visit and baseline were evaluated using the Friedman test, followed by a two‐tailed, pairwise comparison using the Wilcoxon signed‐rank test with post‐hoc Bonferroni correction. A Kaplan–Meier curve was constructed to estimate the probability of symptom non‐relapse rate after termination of the DMSO therapy. Logistic regression analysis of baseline characteristics was applied to identify factors predictive of treatment response at 12 weeks. A *p* value of <0.05 (or 0.0033 for multiple comparisons) was considered statistically significant. All statistical analyses were calculated by JMP®, version 14 (SAS Institute). Data are expressed as the mean ± standard deviation (SD).

## RESULTS

### Patients

A total of 35 patients with HIC (including 21 females) received the standard intravesical DMSO therapy during the study period. Among them, five patients were excluded from the study due to the lack of complete clinical data (*N* = 3), parallel use of oral PSL (*N* = 1), and concomitant chemotherapy for comorbid hematological malignancy (*N* = 1). The remaining 30 patients (mean age, 68.3 ± 12.2) (including 18 females), who completed the GRA and symptom questionnaires and provided 24‐h frequency volume charts at every follow‐up visit were included in further analysis. The demographic and baseline characteristics are shown in Table 1. Patients had undergone a mean number of 2.0 ± 1.2 (range, 1–6) sessions of transurethral electrocautery of Hunner lesions with bladder hydrodistension, and six of them had also received oral PSL treatment at the time of symptom relapse after surgery. Another three patients had undergone intravesical injection of heparin and alkalized lidocaine (*n* = 2), or BoNT‐A (*n* = 1), as a salvage therapy after endoscopic surgery. Previous medicines are listed in Table 1. Among these, opioids, acetaminophen, NSAIDs, and pregabalin were allowed during DMSO treatment.

### Treatment outcomes of DMSO therapy

**FIGURE 1:**
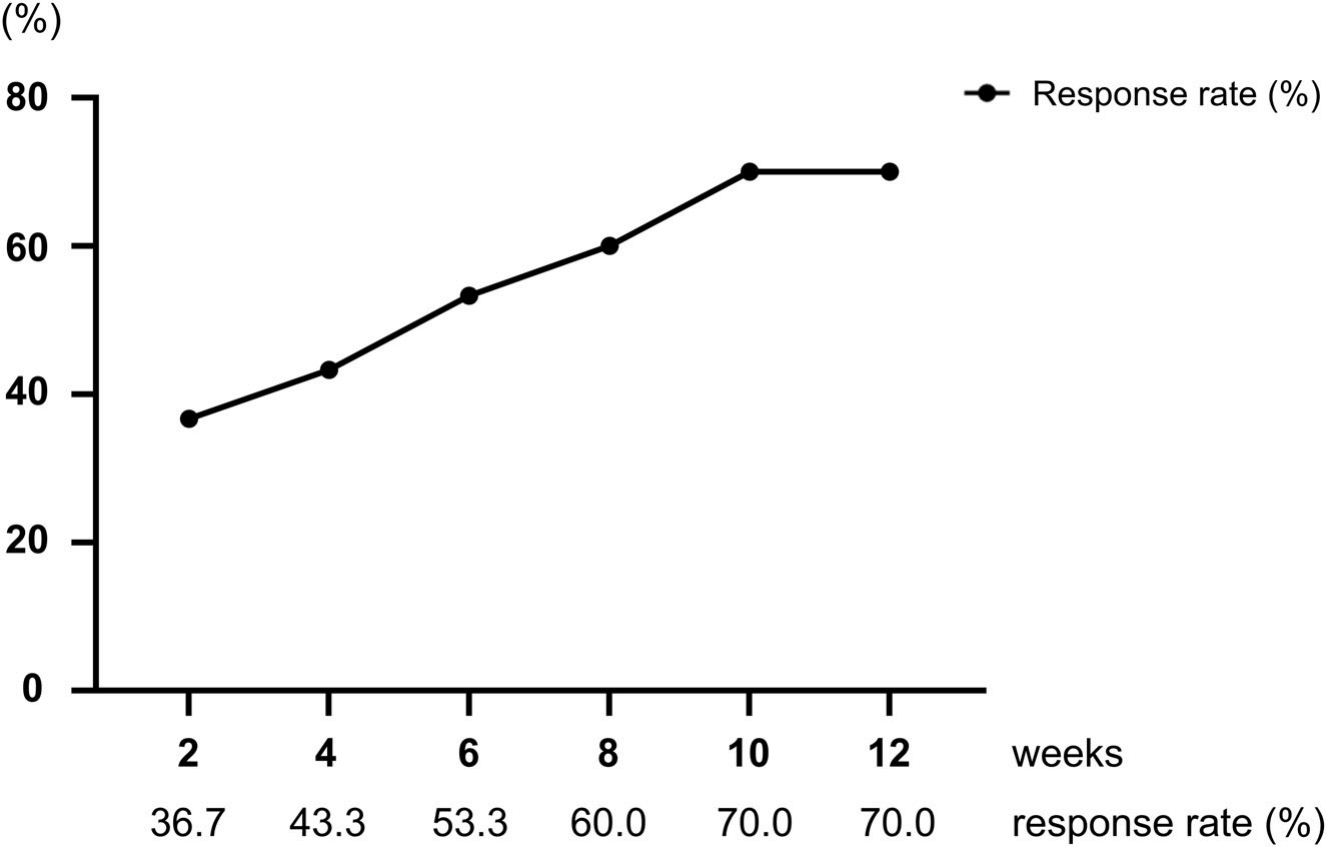
Overall response rates during the dimethyl sulfoxide (DMSO) treatment. Treatment response was evaluated using a 7‐graded global response assessment (GRA) questionnaire (markedly improved (+3), moderately improved (+2), slightly improved (+1), no change (0), slightly worse (−1), moderately worse (−2), and markedly worse (−3)). Patients who rated the efficacy as better than +2 in the GRA were defined as responders. DMSO was administered at 0, 2, 4, 6, 8, and 10 weeks.

**FIGURE 2:**
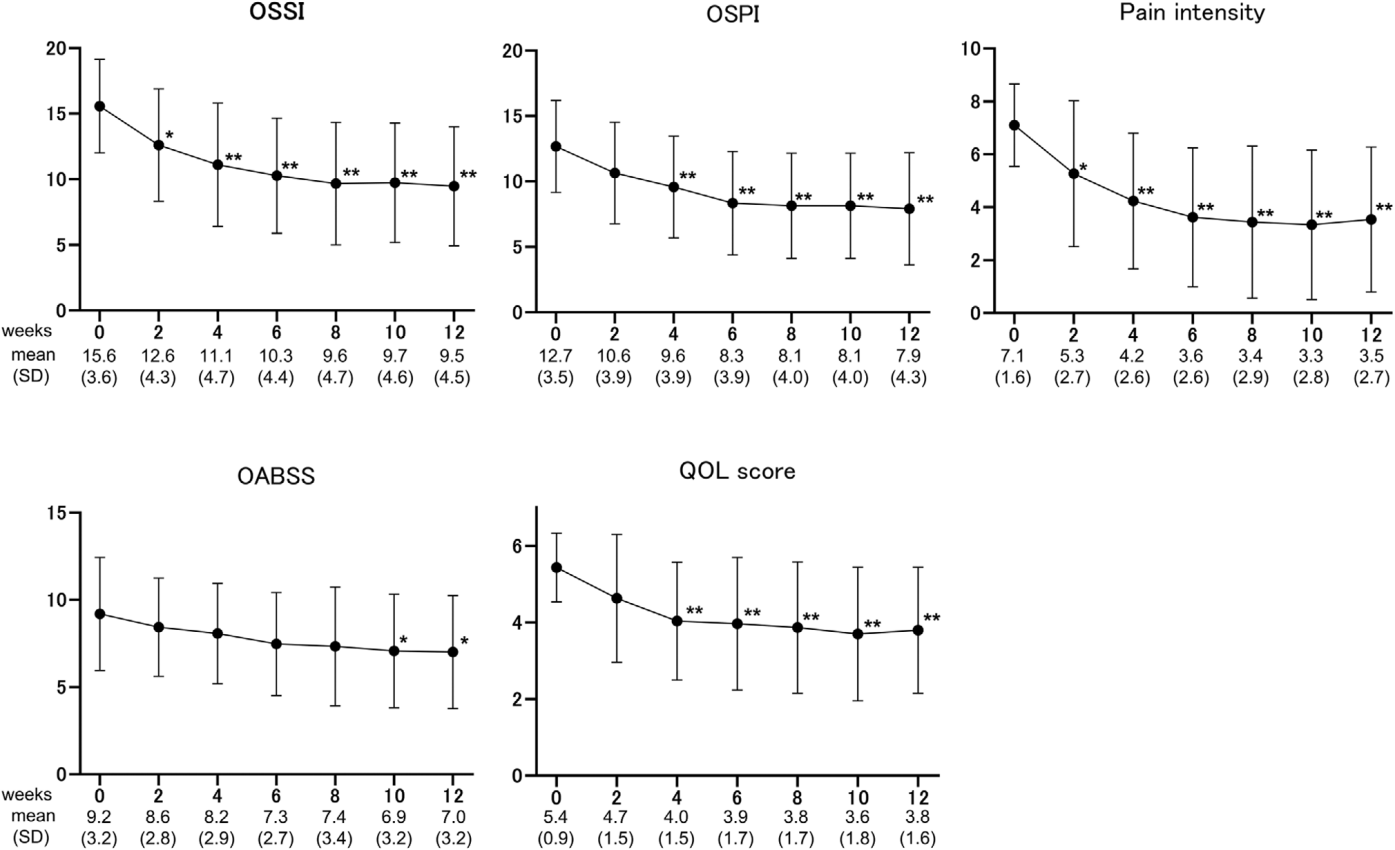
Symptom and QOL parameters during the DMSO treatment. The OSSI/OSPI scores, pain intensity, and QOL score improved significantly at 4 weeks posttreatment initiation, and efficacy was maintained during the treatment period. The OABSS score improved significantly after 10 weeks of treatment. Values are expressed as the mean ± standard deviation (SD). *p < 0.001, **p < 0.0001, statistically significant difference between each visit and baseline (0 weeks); two‐tailed, pairwise comparison conducted using the Wilcoxon‐signed rank test with post hoc Bonferroni correction. DMSO, dimethyl sulfoxide; OABSS, overactive bladder symptom score; OSSI/OSPI, O'Leary and Sant symptom index/O'Leary and Sant problem index; PSL, prednisolone; QOL, quality of life.

**FIGURE 3:**
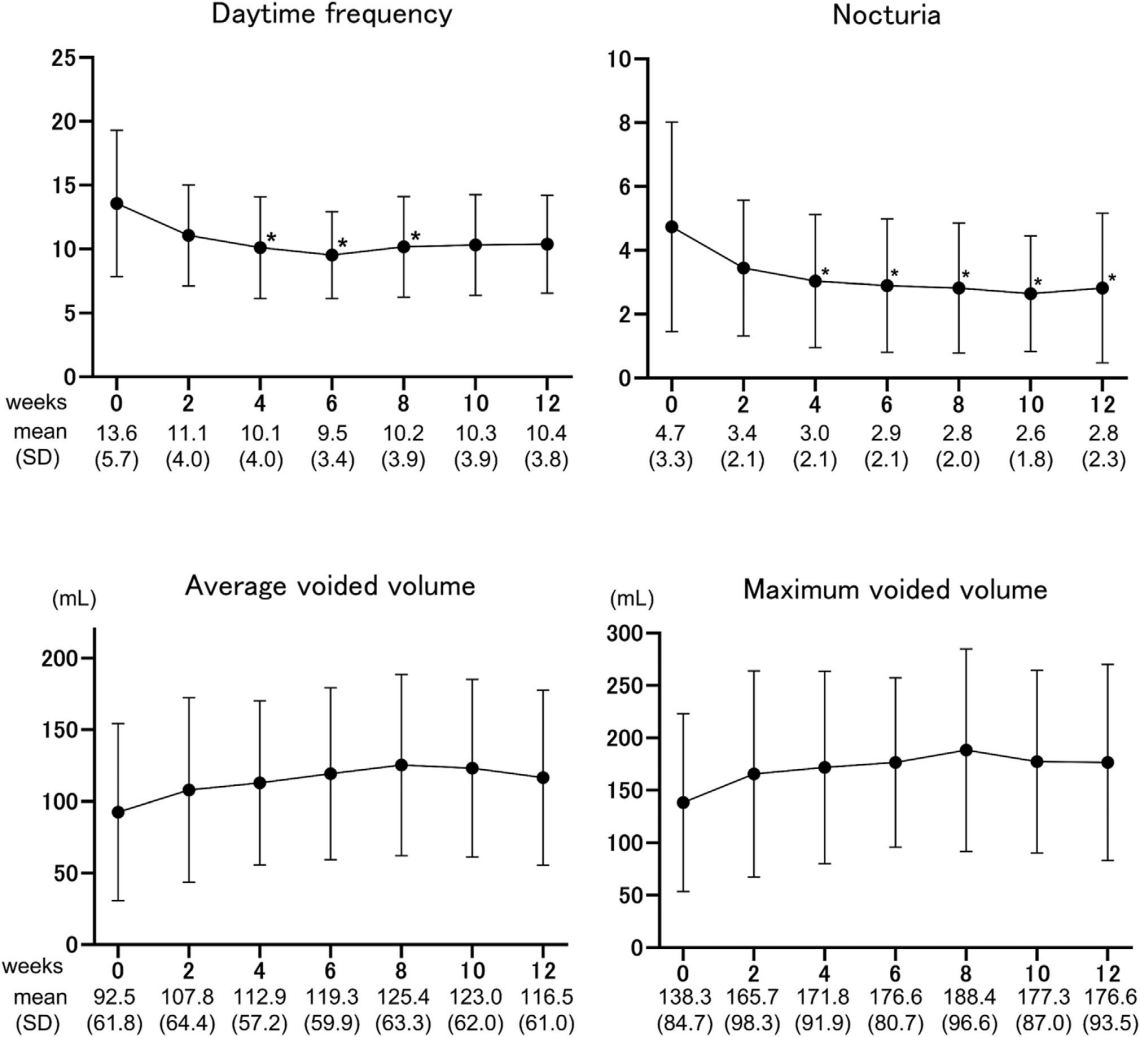
Urinary frequency and voided volume during dimethyl sulfoxide (DMSO) therapy. Nocturnal urinary frequency was reduced significantly after 4 weeks of DMSO treatment, while daytime frequency was reduced significantly at 4, 6, and 8 weeks. The average and maximum voided volume increased gradually during the treatment period, but the increase was not significant. *p < 0.001, **p < 0.0001; statistically significant difference between each visit and baseline (0 weeks). A two‐tailed, pairwise comparison was conducted using the Wilcoxon‐signed rank test with post hoc Bonferroni correction. Values are expressed as the mean ± standard deviation (SD).

All analyzed patients completed all six instillations of DMSO for 12 weeks. Of these, three received half a dose of DMSO with a shortened dwell time in the bladder (5–10 min). The overall response rates at 2, 4, 6, 8, 10, and 12 weeks were 36.7%, 43.3%, 53.3%, 60.0%, 70.0%, and 70.0%, respectively (Figure 1; Figure S1). Compared with baseline (Week 0) values, the OSSI/OSPI scores, pain intensity, urinary frequency, and QOL score fell significantly after 4 weeks of treatment. Improvement of the OSSI/OSPI scores, pain intensity, nocturia, and QOL scores was maintained over the course of treatment (Figure 2). Meanwhile, the OABSS score and average and maximum voided volumes showed a tendency toward improvement, although the difference was not significant (Figures 2 and 3). Three responders chose to undergo cystoscopy after treatments. Of these, Hunner lesions disappeared after DMSO therapy in two patients and decreased in one patient (Figure S2). There were no pretreatment parameters predictive of treatment response at 12 weeks (data not shown).

**FIGURE 4:**
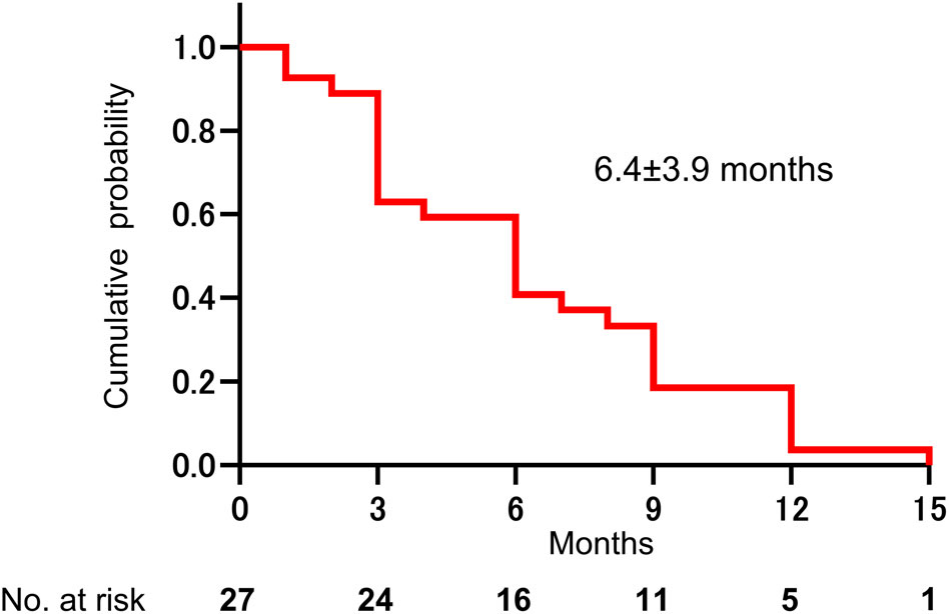
Kaplan–Meier curve shows symptom relapse after dimethyl sulfoxide (DMSO) treatment. Duration to symptom relapse after termination of DMSO treatment was calculated using Kaplan–Meier curves. Patients who rated their condition as better than +1 (slightly improved) in the global response assessment (GRA) at the end of the treatment period (12 weeks) were followed up after DMSO treatment. Symptom relapse was defined when patients rated their condition as worse than −1 (slightly worse) on the GRA. Duration of symptom relief was then measured as the period between 12 weeks and the time of symptom relapse.

Three patients rated their condition as no change (*n* = 1) or worse (*n* = 2) at 12 weeks compared with baseline (Figure S1); of these, the patient with no change requested to rechallenge the second course of the standard DMSO treatment, and the two worsened patients received salvage endoscopic surgery and oral PSL therapy, respectively, after DMSO therapy. For the remaining 27 patients with GRA better than slightly improved (+1), the mean duration to symptom relapse after termination of a single course of DMSO therapy was 6.4 ± 3.9 months (range, 1–15 months) (Figure 4). At symptom relapse, the second course of the standard DMSO treatment (*N* = 12), maintenance DMSO therapy with regular monthly administration (*N* = 3), oral PSL therapy (*N* = 4), endoscopic surgery (*N* = 1), urethral catheterization (*N* = 1), analgesic medication (*N* = 4), was given as a next treatment, while six patients had been observed without any therapies until the end of the study period.

### Safety of the intravesical DMSO therapy

Observed AEs are listed in Table 2. The most common AE was an acute infusion reaction, observed in 21 patients (70.0%); this included bladder/urethral irritation and urinary frequency/urgency (within 3 days), with or without reactive incontinence. No patients complained of the characteristic breath odor associated with DMSO. All side effects were tolerable after premedication with a diclofenac suppository, and/or by adjusting the infusion dose and dwell time in the bladder; no patient ceased DMSO treatment due to the AE.

**TABLE 2:** Adverse events after dimethyl sulfoxide treatment.

Adverse event	No. of patients (%)
Acute infusion reaction (≤3 days)	21 (70.0)
Bladder/urethral irritation	19 (63.3)
Urinary frequency/urgency	8 (26.7)
Reactive incontinence	6 (20.0)
Lower urinary tract symptoms (≥4 days)	2 (6.7)
Bladder/urethral pain/discomfort	2 (6.7)
Urinary frequency	2 (6.7)

## DISCUSSION

Here, we present real‐world data related to the recently approved standardized intravesical DMSO therapy regimen for patients with HIC in Japan. A total of six consecutive treatments with DMSO led to a significant improvement in bladder pain, IC/BPS symptom scores, urinary frequency, and QOL score, without causing serious AE. The therapeutic efficacy was maintained beyond the treatment period (a mean 6 months of symptom relief after termination of treatment). Of note, the efficacy of the current DMSO treatment was highlighted by marked pain relief, which is the hallmark and most troublesome symptom of HIC, and thus DMSO treatment might contribute to a significant improvement in QOL.

To date, endoscopic electrocautery of Hunner lesions or systemic immunomodulatory therapies using Cyclosporine A or corticosteroids have been used as treatments for HIC.^1^, ^18^ However, frequent electrocautery of Hunner lesions carries the risk of bladder malformation and reduced bladder capacity. Recently, we showed that maximum bladder capacity at a pressure of 80 cm H_2_O could fall by approximately 50 mL per single session of electrocautery of Hunner lesions.^19^ Given this risk, electrocautery of Hunner lesions should be performed as few times as possible. In the present study, the participants had undergone this endoscopic surgery a mean of 2.0 times, and their bladder capacity had already decreased to some degree, with a mean volume of less than 400 mL at hydrodistension. Lim et al. showed that reduced bladder capacity was a significant predictive factor for DMSO treatment failure.^13^ Our finding that voided volumes did not improve as significantly as pain and QOL, which has been also reported in some past studies,^5^, ^6^, ^9^ may be explained by this reduced bladder capacity. By contrast, immunomodulatory drugs can be associated with significant AE such as susceptibility to severe infection, malignancies, renal dysfunction, hypertension, and glucose intolerance.^20^, ^21^, ^22^, ^23^ These serious complications mean that many urologists are not willing to use immunosuppressants to treat HIC in routine practice. Thus, alternative, safe, conservative treatments for HIC are needed.

With respect to this, a variety of intravesical treatments have been implemented for patients with HIC to minimize AE. Previous studies report that injection of a corticosteroid into the bladder wall at the site of Hunner lesions, or injection of BoNT‐A into the bladder trigonal wall, are effective for HIC.^24^, ^25^ However, these procedures require operating space and special devices usually with general/local anesthesia and are accompanied by the possible risk of bleeding at the injection sites of the bladder wall. Furthermore, the efficacy of these treatments is relatively short (about 6 months), and most patients need repeat procedures to maintain the therapeutic effects, which imposes a healthcare cost burden on both patients and the medical system. Previously, we also tested the efficacy of 12‐weekly intravesical instillations of a combination of heparin and alkalized lidocaine to treat patients with IC/BPS.^26^ The treatment efficacy was, however, relatively poor compared with that of DMSO, with a maximum response rate of 20.0% during the treatment period; also, efficacy lasted for only 2 months after the last instillation. As for DMSO, other studies reported that the efficacy of intravesical DMSO treatment was comparable to that of other agents such as *Mycobacterium bovis* bacillus Calmette–Guérin, amide local anesthetics, corticosteroids, and heparin‐like substances.^9^, ^12^, ^13^ Of note, past evidence suggests that a combination of DMSO plus other such agents does not provide additional benefits over DMSO alone.^27^ In the present study, we observed that the duration of symptom relief after termination of a single course of the standard DMSO therapy in Japan was a mean of 6.4 months, which is equivalent to that provided by intravesical BoNT‐A treatment (mean 5.4 months) previously used for Japanese patients with refractory HIC.^25^ Thus, intravesical DMSO therapy is a safe, cost‐effective, and convenient treatment option for both patients and physicians, and the benefits are comparable with those of other intravesical therapies. Further prospective, randomized comparative studies of DMSO and other intravesical treatments are needed to verify these data and compare them with those obtained for other established intravesical treatments for HIC.

To date, there are no clear indications regarding the appropriate treatment regimen for intravesical DMSO treatment for HIC. In the past, most studies administered 50 mL DMSO weekly or every 2 weeks for a total of 3–6 instillations, with less than 20 min of dwell time at instillation.^27^ In Japan, the standard treatment protocol for intravesical DMSO therapy for HIC proposed at the time of Ministry approval was based on previous studies: instillation every 2 weeks for 12 weeks, with a total of six instillations. Here, we performed intravesical DMSO treatment according to this standardized regimen for patients with refractory HIC, which revealed that it was effective and safe, as reported previously in a randomized prospective study.^16^ Thus, our study provides a reliable treatment regimen for intravesical DMSO therapy for refractory HIC. However, the duration of the efficacy of this regimen was relatively short, with a mean of 6.4 months. Given the chronic disease nature of HIC, many patients will require repetitive treatments with this standard DMSO regimen. With regard to this, Tomoe reported that DMSO administration as a maintenance adjuvant therapy after endoscopic surgery, comprised of weekly instillations for 8 weeks, every 2‐week instillations for 16 weeks, and every 4‐week instillations thereafter, significantly improved the OSSI/OSPI scores, pain intensity, and bladder capacity over 18 months in patients with HIC, compared with surgery alone.^28^ Future evaluations are warranted to find the optimal treatment strategies of intravesical DMSO therapy including strategies employing combinations of the standardized regimen and subsequent maintenance administration, to increase the long‐term symptom relief and tolerability of DMSO in patients with refractory HIC.

This study has several limitations. First, the retrospective nature of the study design and relatively small sample size, along with the potential placebo effect owing to consistent follow‐up by a single urologist (YA), may limit the methodological quality. Second, the lack of a control group limits the interpretation of the demonstrated efficacy of DMSO therapy. Further accumulation of evidence regarding intravesical DMSO treatment for HIC is warranted to assure the development of appropriate treatment protocols and to maximize the therapeutic benefits of this cost‐effective, safe, and easy‐to‐handle drug.

In conclusion, we show that the recently approved, standardized intravesical DMSO therapy in Japan is an effective, safe, and moderately tolerable treatment for patients with refractory HIC, particularly in the context of reduced pain and improved QOL.

## AUTHOR CONTRIBUTIONS

**Yoshiyuki Akiyama:** Conceptualization; methodology; software; data curation; formal analysis; investigation; funding acquisition; visualization; writing—original draft; project administration. **Aya Niimi:** Supervision. **Akira Nomiya:** Supervision. **Satoru Taguchi:** Supervision. **Yuta Yamada:** Supervision. **Yusuke Sato:** Supervision. **Taketo Kawai:** Supervision. **Daisuke Yamada:** Supervision. **Haruki Kume:** Supervision. **Yukio Homma:** Supervision; writing—review & editing; funding acquisition.

## FUNDING INFORMATION

This study was supported by a KAKENHI Grants‐in‐Aid from the Japan Society for the Promotion of Science (JSPS) (grant number 22K16788 to YA) and a Health Labour Sciences Research Grant from the Ministry of Health, Labour, and Welfare (grant number 18060798 to YH).

## CONFLICT OF INTEREST STATEMENT

The authors have no relevant financial interests to disclose regarding the materials discussed in the manuscript.

## APPROVAL OF THE RESEARCH PROTOCOL BY AN INSTITUTIONAL REVIEWER BOARD

The study protocol, including the use of an opt‐out methodology for gaining informed consent, was approved by the institutional review board of the University of Tokyo (approval no. 3124) and it conforms to the provisions of the Declaration of Helsinki.

## INFORMED CONSENT

Participants were informed about this study using generally accessible contact information. Written informed consent was obtained from all patients who chose to participate in this study. All procedures followed appropriate guidelines.

## REGISTRY AND THE REGISTRATION NO. OF THE STUDY/TRIAL

N/A.

## ANIMAL STUDIES

N/A.

## Supporting information


Figure S1.
