# Supplementary material for: Efficacy and safety of intravesical dimethyl sulfoxide treatment for patients with refractory Hunner‐type interstitial cystitis: Real‐world data postofficial approval in Japan
Source: Int J Urol. 2023 Oct 11;31(2):111–8. doi: 10.1111/iju.15320 (PMC11524091; doi:10.1111/iju.15320)
Supplement: Supplementary file 1 — Figure S1. [file IJU-31-111-s001.docx]

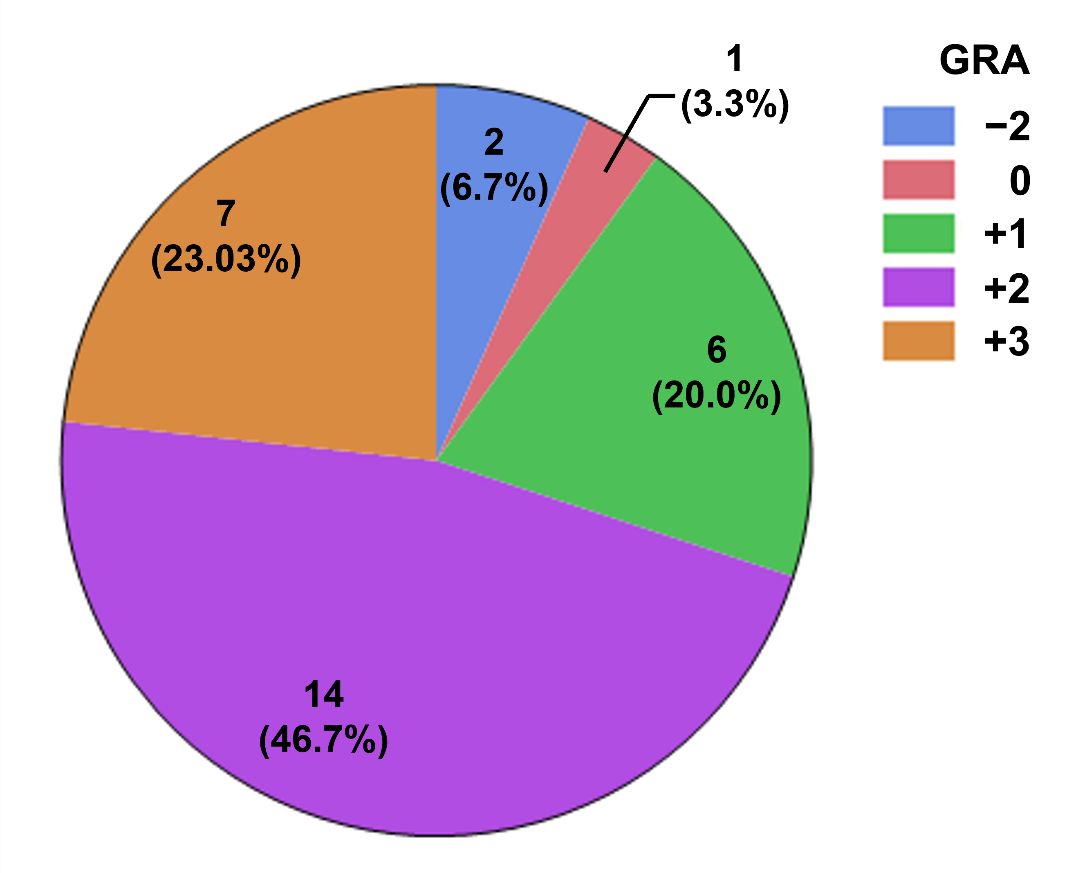
**Supplementary Figure S1.** Treatment response after 12 weeks of DMSO therapy

Numbers in each segment indicate the number (%) of patients.

GRA (global response assessment): markedly improved (+3), moderately improved (+2), slightly improved (+1), no change (0), slightly worse (-1), moderately worse (-2), and markedly worse (-3).

**Supplementary Figure S2.** Representative cystoscopic images showing disappearance of Hunner lesions after DMSO treatment.


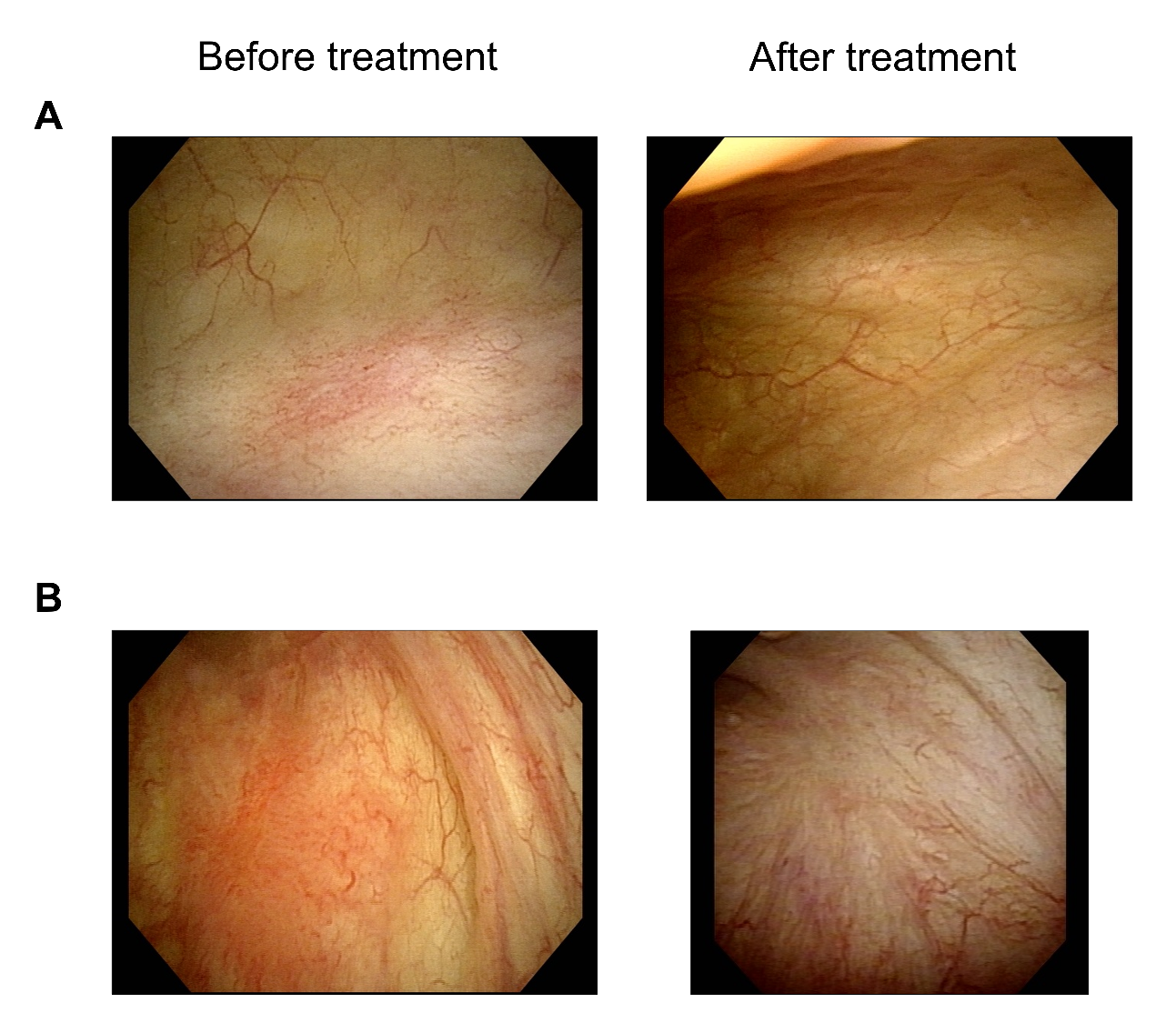


A. An 84-year-old female patient with HIC.

(Left) Hunner lesions in the left-lower-posterior wall, observed before DMSO treatment.

(Right) Disappearance of the Hunner lesions at 12 weeks after six injections of intravesical DMSO.

B. A 71-year-old male patient with HIC.

(Left) Hunner lesions in the left-lateral wall, observed before DMSO treatment.

(Right) Disappearance of the Hunner lesions at 12 weeks after six injections of intravesical DMSO.

DMSO: dimethyl sulfoxide; HIC: Hunner-type interstitial cystitis.
